# The human impact on North American erosion, sediment transfer, and storage in a geologic context

**DOI:** 10.1038/s41467-020-19744-3

**Published:** 2020-11-26

**Authors:** David B. Kemp, Peter M. Sadler, Veerle Vanacker

**Affiliations:** 1 State Key Laboratory of Biogeology and Environmental Geology, School of Earth Sciences, China University of Geosciences, Wuhan, 430074 P.R. China; 2 Department of Earth Sciences, University of California, Riverside, CA 92521 USA; 3 Georges Lemaitre Centre for Earth and Climate Research, Earth and Life Institute, Université catholique de Louvain, Louvain-la-Neuve, Belgium

**Keywords:** Environmental impact, Geomorphology, Sedimentology

## Abstract

Humans are primary agents of geomorphic change, and rates of anthropogenic landscape change likely far exceed the pace of change expected from natural geologic processes. Nevertheless, our understanding of the impact of humans on the natural landscape is limited by difficulties in accurately comparing past and present rates of change across wide spatial and temporal scales. Here, we present a compilation of  >4000 rates of alluvial sediment accumulation that provide an indirect record of North American erosion, mass transfer and sediment storage from the late Pleistocene to the present day. Continent-wide rates of alluvium accumulation were broadly stable for ~40,000 years, but increased 10-fold during the rapid expansion of agriculture and river system modification associated with European colonization. Interpreted in terms of mass transfer, humans have moved as much sediment in North America in the past century as natural processes can transfer in 700–3000 years.

## Abstract (web summary)

Human activities have accelerated soil erosion and landscape change in many areas. Here the authors show how rates of erosion, sediment transfer and alluvial sedimentation have increased by an order of magnitude across North America since European colonization, far exceeding the rates expected of natural processes.

## Introduction

Human activities have significant impacts on landscape evolution via changes in sediment production, transport and storage^1–6^. In particular, agricultural practices such as soil tillage and deforestation increase soil erosion rates, river sediment loads, and landslide susceptibility^7,8^. At the same time, human modification of streams and rivers, particularly by damming, alters channel morphology and flow regime, with consequent impacts on floodplain environments and sediment storage^3,5^. In a geologic context, such changes are likely unprecedented^4^. Notably, soil losses as a consequence of human activities likely exceed continental denudation rates of the last 0.5 billion years of Earth history^4,9^. Nevertheless, quantifying both past and present rates of geomorphic change has proven challenging and controversial^10–15^. There are intrinsic limitations in our ability to accurately compare past and present rates of change across temporal and spatial scales^15,16^, although this is a critical prerequisite for contextualizing anthropogenic impacts.

The influence of humans on alluvial sedimentation is well established^3–5,17,18^, and analysis of alluvial deposits provide an indirect means to ascertain geomorphic changes such as accelerated soil erosion and changes in channel/floodplain sediment storage linked to human activity. Soil erosion plot-scale studies have shown that land use and cover have a dominant influence on soil losses, with erosion rates on arable and bare land that may exceed rates of soil formation by as much as two orders of magnitude^19,20^. Owing to the development of intensive farming practices and industrialization, the impact of land and water management practices on soil erosion and alluvial sedimentation are often readily observable, particularly in North America^1,3,4,6,17,21,22^. There, alluvial deposits associated with changes in land- and water-use (“post-settlement alluvium”) were typically deposited at faster rates than pre-settlement strata (e.g., refs. ^1,4,17,21^). Previous work to understand the impact of humans on alluvial systems and the magnitude of post-settlement increases in erosion and sedimentation has typically focused on individual catchments and often are conducted across relatively narrow timescales. These spatial and temporal limitations mean that it is difficult to determine human impacts on landscape evolution at scales representative of entire continental areas, and at timespans sufficient to contextualize human impacts against long-term natural (i.e., geologic) background variability.

In this study, we have used a continent-scale compilation of 4754 alluvium accumulation rate measurements from 400 study sites in North America to establish the timing, pattern and long-term geologic context of alluvium accumulation linked to anthropogenic landscape changes across the past ~40 k.y. (i.e., late Pleistocene to modern).

## Results and Discussion

### Late Pleistocene to modern alluvium accumulation rates in North America

**Fig. 1:**
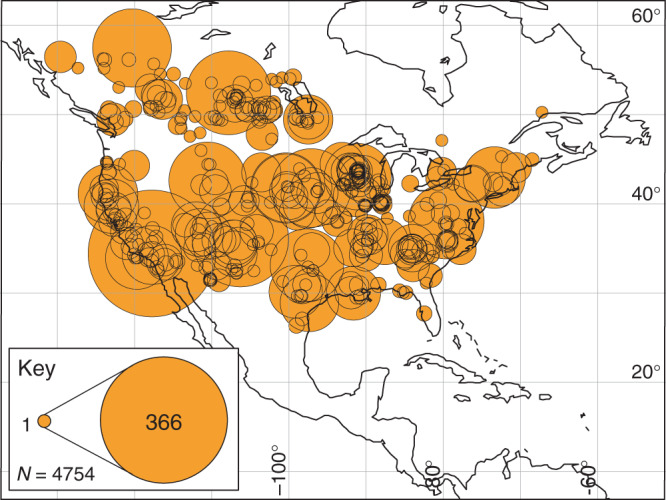
Bubble plot showing distribution of alluvium accumulation rate data in the compilation. Bubbles indicate the study sites (400 in total) and the number of rate measurements from each site (4754 in total). See Supplementary Data 1 for full data listing.

**Fig. 2:**
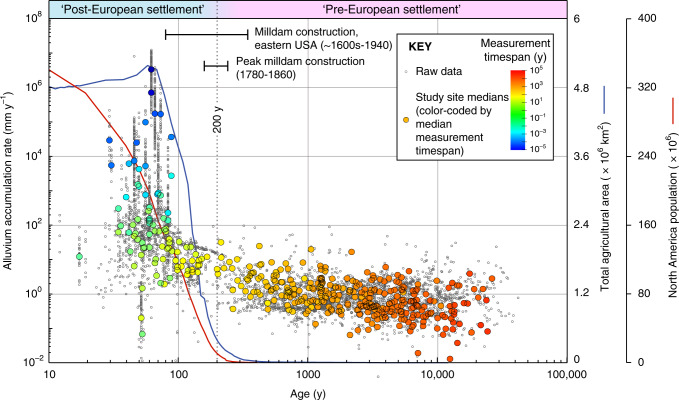
North American alluvium accumulation rates versus age. Raw data are shown as gray circles (note logarithmic scales). Colored datapoints are the median rates and ages at each study site, with color-coding based on the timespan of measurement. Note the tendency for rates measured over short timespans to be faster (particularly apparent in rates aged <100 y), illustrating how rates are partially biased by timespan dependence (see text and Fig. 3a for details). Nevertheless, there is a stepwise increase in rates between the approximate pre- and post-settlement time periods ~200 y ago (~1820 CE). This increase coincides with a marked expansion in total agricultural land area in North America (blue line, data from ref. ^23^) and the onset of rapid population growth (red line, data from ref. ^45^. USA population prior to 1790 includes only white Europeans^45^). Agriculture and population increases are bracketed by an interval of intensive milldam construction in both Canada and USA (dates from refs. ^5,24,25^). The stepwise increase in accumulation rates ~200 y ago is not attributable solely to timespan dependence, and reflects an anthropogenic impact on alluvium sedimentation.

**Fig. 3:**
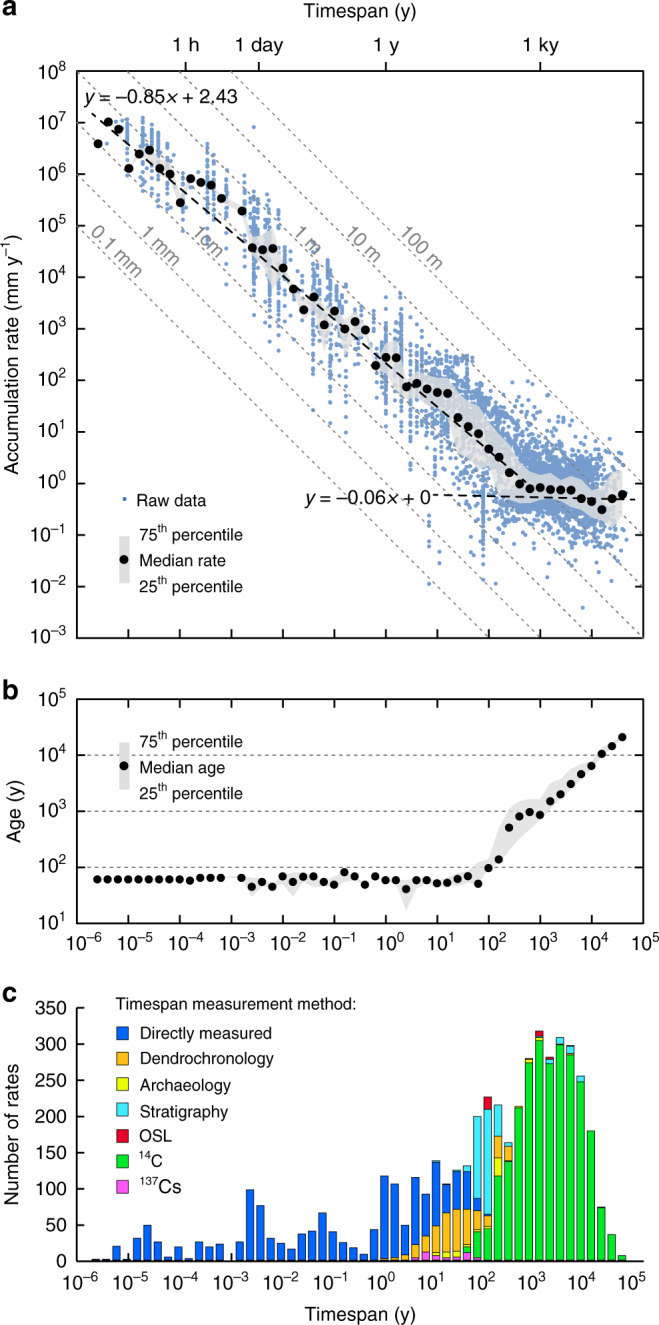
Timespan and age dependence on alluvium accumulation rates. **a** Timespan versus rate plot for all data in the compilation (blue circles). Median rates (black circles) and associated 25–75th percentile range (gray shading) within 0.2 log timespan bins also shown. Gray dashed lines are contours of constant sediment thickness. **b** Timespan versus age plot. Median ages are shown as black circles, and associated 25–75th percentile range is marked by gray shading (0.2 log timespan bins). **c** Histogram showing data abundance in each 0.2 log timespan bin, and the range of timespan dating methods used for calculating rates. See Methods for information on these measurement methods.

Figure 1 shows the distribution of North American alluvium accumulation rates in the compilation, and Fig. 2 shows these rates plotted against age. Rates are defined as sediment thickness divided by the timespan of accumulation, with timespan measured using a variety of methods: e.g., direct measurement of active sedimentation, and geologic dating of previously deposited sediments (for instance, ^14^C, dendrochronology; see Methods and Fig. 3). Rates are highly variable, spanning 8 orders of magnitude (from <10^−2^ mm y^−1^ to >10^6^ mm y^−1^). Ages range from 41 k.y. BP to 2007 CE. Between 41 k.y. and ~200 y ago, the median accumulation rate is 0.7 mm y^−1^, and rates show relatively little variation with age. At ~200 y there is a stepwise increase in accumulation rates, and the median value of rates younger than 200 y is ~2 orders of magnitude faster than pre-200 y rates (median 75 mm y^−1^) (Fig. 2). Change-point and rank sum analysis confirm that rates change most markedly between ~100 and ~300 y ago (i.e., ~1720–1920 CE), with a median of ~200 y (~1820 CE) (see Methods). This age range is broadly coincident with rapid population growth driven by European colonization (Fig. 2). Closely linked to this colonization was a marked expansion of agricultural land use that began ~1700 and peaked in 1960 (Fig. 2)^23^. Total agricultural land area increased from <0.1 × 10^6^ km^2^ to 5.2 × 10^6^ km^2^ over this time interval, before declining slightly to a present day value of 4.7 × 10^6^ km^2^ (Fig. 2)^23^. Similarly, population and agriculture growth occurred across a period that was also marked by extensive construction of dams for the nascent milling industries of North America, notably in the east of both Canada and the USA^5,24,25^ (Fig. 2). River damming to harness water power for milling and associated agricultural and industrial purposes likely peaked in the USA between 1780 and 1860, by which time many thousands (perhaps millions^26^) of dams had been constructed^5^.

### Timespan bias in alluvium accumulation rates

Median rates for individual study sites are shown as colored circles on Fig. 2, with the color-coding based on the median timespan over which rates were measured. These data emphasize how the increase in accumulation rates ~200 y ago is observed in multiple studies covering a wide range of averaging timespans and diverse environmental settings. Importantly however, these data also emphasize how rates of sediment accumulation are partly dependent on the timespan of measurement: fast rates in the compilation are associated with short measurement timespans, and slow rates are associated with long timespans (Fig. 2). Rates in the compilation, like those typically observed in other sedimentary environments, increase with decreasing measurement timespan as a power law^16,27,28^ (Fig. 3). This effect constitutes a significant bias that arises largely because of the episodic and erosive nature of the sedimentation process, which means that longer timespans inevitably include more and longer erosive events and hiatuses^16^. In addition, observational biases in sediment accumulation rate measurements may result in a tendency to measure unusually extreme sedimentation at the present day, and not intervals of quiescence^28^.

In our compilation, timespan bias exerts the dominant control on rates of alluvium accumulation (Fig. 3). Measured rates of accumulation fall by ~0.9 orders of magnitude for every 1 order of magnitude increase in measurement timespan, regardless of how rates are measured (Fig. 3, see also Supplementary Fig. 1). In detail, at timespans longer than ~1000 y the scaling shallows to a slope of ~0, reflecting how at longer timespans sediment preservation in alluvial sinks is promoted by burial, and the risk of erosion of sediment piles *>*~1 m thick is low (Fig. 3, see also ref. ^27^). Age and timespan are coupled at timespans >100 y, with age scaling as an apparent power law with increasing timespan of measurement (Fig. 3).

Taken together, in order to accurately quantify and compare changes in accumulation rates through time, and thus confirm the veracity and magnitude of the stepwise increase in alluvium accumulation rates ~200 y ago coincident with European colonization, we have statistically compared rates measured at comparable timespans either side of this age.

### Direct comparison of pre- and post-settlement accumulation rates

**Fig. 4:**
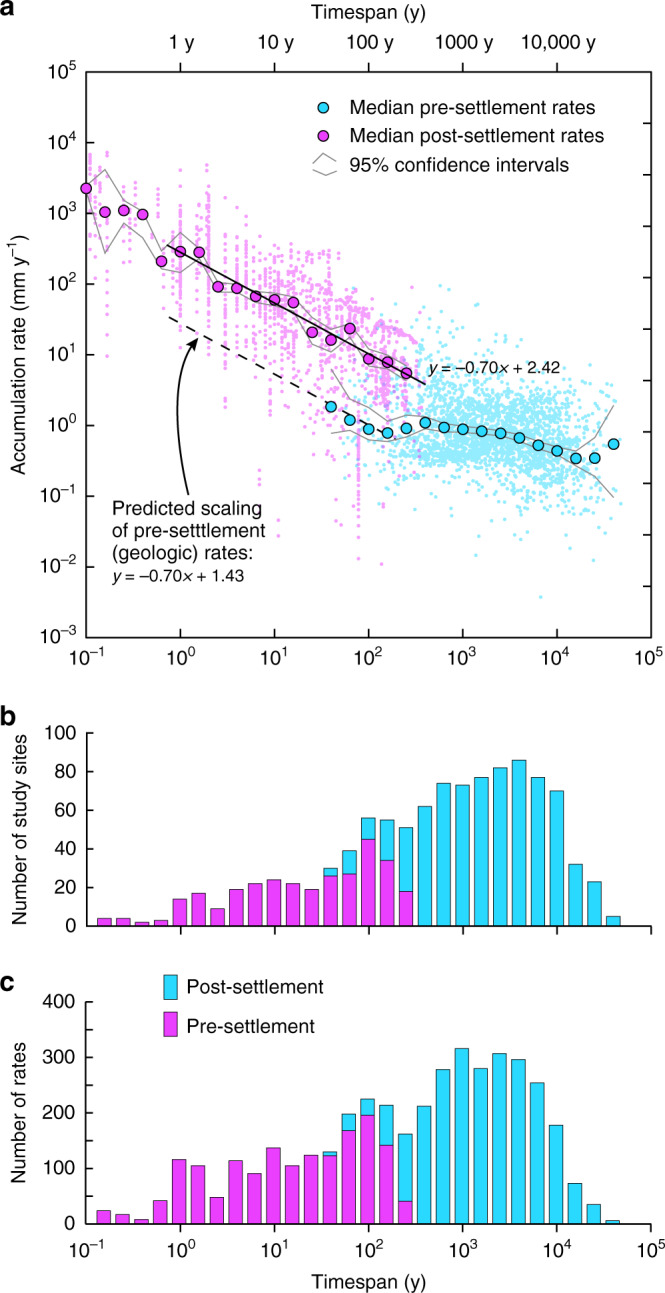
Alluvium accumulation rates versus timespan from 0.1 y to 40 k.y. **a** Plot of alluvium accumulation rates versus measurement timespan across timespans from 0.1 y to 40 k.y. Data are divided into pre- and post-settlement (ages >200 y and <200 y, respectively). Raw data are shown as small colored circles. Larger filled circles are the median rates for pre- and post-settlement data in 0.2 log timespan bins. Pre- and post-settlement rates co-occur in timespan bins 1.6–2.4 log years (i.e. ~40–400 y). Note how at these timespans median post-settlement rates are consistently ~10× faster than median pre-settlement rates. Medians are based on Monte Carlo modeling of the raw data that takes into account timespan measurement errors. Gray lines show the 95% confidence intervals associated with this error modeling. See Methods and main text for details. Regression line and equation through the post-settlement data highlights the clear timespan dependent scaling of post-settlement rates between timespans of 1 and 400 y. The dashed regression line through the pre-settlement data and accompanying equation shows the predicted scaling of pre-settlement (i.e., natural geologic) rates at timespans from 1 to ~40 y. This predicted scaling is used to estimate the difference between post-settlement rates and the expected pre-settlement (i.e., natural geologic) rate of accumulation at timespans between 1 and 40 y. **b** Histogram showing number of study sites within each 0.2 log timespan bin. **c** Histogram showing counts of pre- and post-settlement rates within each timespan bin.

Figure 4 shows rates of alluvium accumulation versus timespan for all rates in the compilation measured at timespans between 0.1 y and 48 k.y. Rates from pre- and post-settlement ages (i.e., older and younger than 200 y, respectively) co-occur at timespans from ~40 to 400 y, and show marked differences (Fig. 4). Within this timespan range, there are 259 pre-settlement rates and 670 post-settlement rates, from a total of 119 separate study sites. Median rates and associated uncertainties within discrete timespan intervals (0.2 log bins) are shown on Fig. 4. These are calculated from Monte Carlo simulations of the raw data that include errors associated with different timespan measurement methods (see Methods). To limit geographic bias caused by the uneven distribution of rate data between study sites (Fig. 1), multiple rates of the same age and timespan from individual study sites were averaged by taking the median before binning. This reduces the total number of rates being compared to 238 pre-settlement rates and 281 post-settlement rates. Median post-settlement rates range from ~6 to ~24 mm y^-1^ at timespans between ~40 and 400 y, whereas median pre-settlement rates at these timespans range from ~0.6 to ~1.2 mm y^-1^ (Fig. 4, Table 1). Overall, median post-settlement rates at timespans between ~40 and ~400 y are ~10× faster than median pre-settlement rates (Fig. 4).

**Table 1:** Key statistics of pre-settlement (age > 200 y) and post-settlement (age < 200 y) alluvium accumulation rates at timespans between ~40 and 400 y (see also Fig. 4).

Log timespan bin (Fig. 4)	Approx. time span range (y)	Pre-settlement rates	Pre-settlement study sites	Raw (and Monte Carlo) pre-settlement median rate (mm y^−1^)	Post-settlement rates	Post-settlement study sites	Raw (and Monte Carlo) post-settlement median rate (mm y^−1^)	Raw rank sum *p* value	% of significant Monte Carlo sims. (*p* value <0.05)^a^
1.6	40–63	7	4	1.21 (1.81)	123	26	15.12 (15.95)	0.0056	90%
1.8	63–100	30	12	1.16 (1.17)	168	27	23.61 (23.15)	<0.0001	100%
2	100–158	29	11	0.68 (0.87)	196	45	6.40 (8.63)	<0.0001	100%
2.2	158–250	72	21	0.59 (0.77)	142	34	7.66 (7.73)	<0.0001	100%
2.4	250–400	121	33	0.68 (0.89)	41	18	5.7 (5.39)	<0.0001	100%

^a^The percentage of Monte Carlo simulations (sims) that yield significant differences between pre- and post-settlement rates (*p* values <0.05).

Wilcoxon rank sum tests indicate that the observed differences in pre- and post-settlement rates are statistically significant (*p* values <0.006) (Table 1, see Methods). These results are not strongly dependent on our choice of 200 y as the cutoff between pre- and post-settlement rates, and we obtain similar results using a cutoff age of 300 y (which our change detection analysis suggests is the oldest age at which rates change significantly in the compilation, see Methods and Supplementary Table 1). Results are also not significantly influenced by the different timespan dating methods used, and rates determined by different methods all show similar increases at ~200 y (Supplementary Fig. 1). We note in particular that radiocarbon (^14^C) dating of wood dominates pre-settlement rate measurements, and a known issue with ^14^C dates is that they can be overestimated (and hence rates potentially underestimated) owing to time lags between wood growth and eventual deposition (the “old wood” effect^29^). Importantly, however, our analyses indicate no evidence of a strong old wood effect in the data (see Methods and Supplementary Fig. 2). Similarly, burial and compaction of older pre-settlement strata is unlikely to amplify pre- and post-settlement rate differences. Even the oldest pre-settlement data are not buried to >~100 m (Fig. 3), and compaction of typical alluvial sediments at depths of up to 100 m will only reduce thicknesses by <3%^30^. The geographic spread of the data means that biases linked to elevation and climate are minimized, despite these factors being of potentially high importance in controlling accumulation rates at individual study sites^12,31^ (see Methods and Supplementary Fig. 3). Although climate has varied in North America through the studied time interval^32,33^ (including an increase in precipitation since the early Holocene^33^) there were no clear changes in hydroclimate that can account for the observed continent-wide increase in accumulation rates between pre- and post-settlement ages.

Taken together, our comparison of pre- and post-settlement rates, taking into account timespan dependence effects, revises the ostensible ~2 orders of magnitude difference observed in Fig. 2 (~100×) to ~1 order of magnitude (~10×).

### Quantifying the anthropogenic impact on the North American landscape

The ~10× difference between pre- and post-settlement accumulation rates on the North American continent is readily attributable to European colonization. The accompanying rapid expansion of farmland had well-documented effects on soil erosion^1,2,10,17–19^. Associated environmental disturbances, such as construction, forestry and ranching, would also have increased erosion, runoff and river sediment loads^2,8,17^. At the same time, significant human management of the riverine environment via damming co-occurred with land use change, increasing alluvial storage capacity^3–5^. In contrast, the landscape impact of pre-European indigenous inhabitants on the continent, whilst potentially of local importance^22^, is not resolvable in the data against the natural variability that characterizes the pre-settlement data prior to dispersal of the first settlers ~15 k.y. ago^34^. Although the resolving power of the compilation falls with increasing age as measurement timespans lengthen, the late Pleistocene to ~200 y interval is characterized by broadly stable alluvium accumulation rates, with the slight rise over this interval (Fig. 2) attributable solely to the timespan dependence effect.

**Fig. 5:**
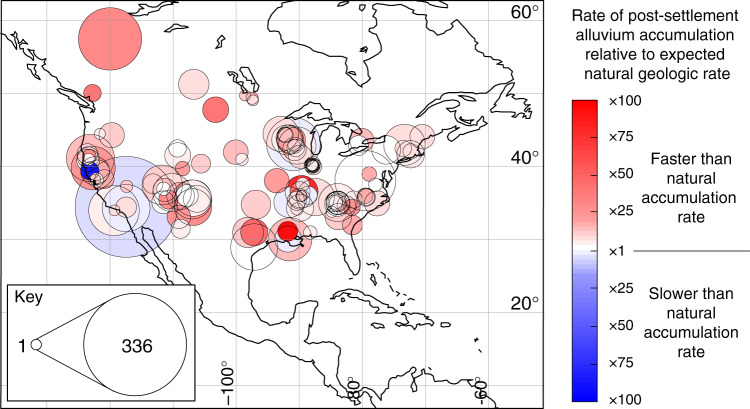
Map showing the distribution and relative magnitude of post-settlement alluvium accumulation rates at timespans between 1 and 400 y. The data are sized according to the number of rates at each site. The color-coding quantifies by how much the median post-settlement rate at the site is faster or slower than the expected rate of natural geologic accumulation measured at the same timespan. The expected natural geologic rate at timespans between 1 and 400 y is defined by the regression slope in Fig. 4. Note how most (94%) of post-settlement rates are faster than the expected natural rate, emphasizing the anthropogenic impact on alluvium accumulation. See main text for details.

To gain further insight into the magnitude and spatial distribution of post-settlement landscape changes relative to natural geologic (pre-settlement) background conditions, we have mapped the difference between pre- and post-settlement accumulation rates (Fig. 5). Because there are no pre-settlement rates in the compilation measured at timespans <40 y, direct comparison of post-settlement accumulation rates and geologic rates on annual to decadal scales is not possible. However, we can predict geologic rates of alluvium accumulation at these short timespans by exploiting the scaling properties of the data. Specifically, we can extrapolate the scaling of pre-settlement rates to timespans shorter then 40 y by assuming that the scaling of these rates would have been the same as that observed for post-settlement rates (Fig. 4). This assumption is supported by the fact that at timespans between ~40 and ~100 y, the scaling of pre-settlement rates has a slope similar to post-settlement rates at the same (and shorter) timespans (Fig. 4). We have not extrapolated pre-settlement rates to timespans <1 y, because at these timespans there are few post-settlement data and slope uncertainty increases (Fig. 4)

Using this predicted measure of pre-settlement accumulation rates at annual to decadal timescales, we can determine the magnitude of post-settlement accumulation rates relative to the expected natural geologic rates for the entire continent at timespans between 1 and ~400 y. Figure 5 is a map of the 126 sites in the compilation where post-settlement accumulation rates have been measured at these timespans. The sites are color-coded to show whether the median post-settlement rate for the site is slower or faster than the expected geologic rate at the same timespan. 94% of sites (119 out of 126) have a median post-settlement rate that is faster than the expected geologic rate for the continent. Of these, 39% (49) have a median rate that is at least 10× faster than the natural rate. Only seven sites (6%) have a median rate of accumulation that is slower than expected of natural processes.

Although the limited number of sites does not represent the full range of environmental variation on the continent, these data exhibit no clear geographic pattern. A significant fraction of the continent is susceptible to erosion linked to land use^4,7^, and as such the wide distribution of high post-settlement rates suggests that anthropogenic landscape erosion and alluvial sedimentation in adjacent watersheds represent continent-wide phenomena. Equally, although 17th–20th century milldams were concentrated in the east of both Canada and the USA^5,24–26^, there are no clear east–west differences in the relative magnitude of the post-settlement rates. Sediment retention by dams was perhaps only of minor importance relative to erosive losses from land use change^4^, and previous work has indicated that most eroded sediment tends to be stored close to its source in any case^4,6,21^.

### Anthropogenic and geologic rates of erosion and sediment transfer

Sadler and Jerolmack^27^ recognized that the timespan dependent scaling of rates of alluvium accumulation is statistically indistinguishable from the scaling of alluvium erosion. This equivalence in scaling, and the fact that log timespan versus log accumulation/erosion rate slopes are close to −1 at short (<10^3^ y) timespans (Fig. 3), emphasizes how episodes of accumulation are effectively balanced by subsequent erosion^27^. Alluvium accumulation rates in our compilation measured over sub-millennial timespans thus reflect sediment mobility in the broad sense and are a proxy for mass transfer. We link these alluvial fluxes to landscape change by assuming, as others have done before^4^, that mass is largely conserved during surficial erosion and the vast majority of post-settlement sediment eroded from the landscape remains in alluvial storage^4,6,21^. Present day soil losses in the USA may exceed ~6000 kg ha^−1^ y^−1^, and were as high as ~9000 kg ha^−1^ y^−1^ a few decades ago^35^. Over the current area of USA agricultural land (4.7 × 10^12^ m^2^, ref. ^23^), this equates to a loss of ~2.8–4.2 × 10^12^ kg y^−1^, and a surficial loss of ~0.5-0.7 mm y^−1^ assuming a dry density of 1250 kg m^−3^ (ref. ^35^). This is close to what was estimated by Wilkinson and McElroy^4^ and references therein (~0.6 mm y^−1^), and also similar to USA cropland erosion estimates compiled by Montgomery^19^, and^10^ Be estimates of USA post-settlement hillslope erosion by Reusser et al.^6^ (~1 mm y^−1^).

At a timespan comparable to the duration of the post-settlement period (i.e., ~200 y), the post-settlement median rate of alluvium accumulation derived from our compilation is ~10 mm y^−1^ (Fig. 4). This long-term post-settlement rate is ~20× higher than the average soil erosion rate. At the annual scale, the median rate of alluvium accumulation over the post settlement period was ~280 mm y^−1^ (Fig. 4), i.e., 400–560× higher than the soil erosion rate. Although clearly highly sensitive to measurement timespan and changes in soil erosion over the past century (e.g., refs. ^10,35^), these results emphasize how the material that is eroded from agricultural land is temporarily stored as alluvium in areas that cover only a small fraction of the agricultural land area^4^. Our long-term estimate of the median rate of post-settlement alluvium accumulation in North America (~10 mm y^−1^) is similar to a previous estimate of alluvium accumulation rate for the post-settlement period (12.6 mm y^−1^) established from 15 locations in the conterminous USA by Wilkinson and McElroy^4^. Although these estimates do not account for sediment that bypasses storage entirely, our results concur with the findings of Wilkinson and McElroy, indicating that alluvial sediment stores cover an area <7% of the agricultural landscape (i.e., <0.34 × 10^12^ m^2^), and are therefore highly responsive to increases in soil erosion owing to human activities.

Relative to these estimates of post-settlement mass transfer, median accumulation rates of pre-settlement alluvium on centennial and longer timescales (up to ~10 k.y.) were broadly consistent, varying between ~0.5 and 1 mm y^−1^ (Fig. 4). If we assume that the total area of alluvial sedimentation has not changed significantly through the Holocene to the present day, this implies an average natural erosion rate of the pre-European conterminous USA landscape (~8 × 10^12^ m^2^) of ~0.01 to 0.04 mm y^−1^ on centennial and longer scales. As noted above, these estimates assume that all eroded sediment is stored as alluvium (and are thus minimum estimates). Locally, natural erosion rates can be faster as areas of high relief and slope are likely to be the major source of naturally eroded sediment^4,31,36^. Our continent-wide erosion rates are within the broad range of geologic rates of soil loss in the USA compiled in Nearing et al.^35^ (~900 kg ha^−1^ y^−1^, i.e. ~0.07 mm y^−1^, but varying between 0.003–0.15 mm y^−1^, assuming dry density of 1250 kg m^−3^). They also bracket the natural average denudation rate estimated for the conterminous USA by Wilkinson and McElroy^4^ (0.021 mm y^−1^), and our minimum estimate is comparable with the estimate in Reusser et al.^6^ (~0.008 mm y^−1^) quantified from 10 large catchments in southeastern USA. On centennial timespans (i.e., comparable to the duration of the post-settlement period), our data imply a natural mass transfer rate of ~0.14–0.42 × 10^12^ kg y^−1^.

Land management changes and restoration efforts since the second world war has helped to lower soil erosion rates in North America in recent decades^10,18,19,35^. Although we have relatively few post-war rates there is some evidence for a fall in alluvium storage over this interval (Fig. 2)—possibly augmented by increased bank erosion of post-settlement alluvium following dam removal and river restoration^5^. Nevertheless, interpreted purely in terms of mass transfer, humans have moved as much material in North America during the last century as would be moved by natural processes in ~700–3000 y.

## Methods

### Data sources and treatment

#### Alluvium accumulation rates

Our compilation of North American alluvium accumulation rates comprises 4754 individual rates from 400 unique study sites (Fig. 1). Data were mined from 183 separate publications. The compilation builds on an earlier version of the compilation published by Sadler^16^ and updated more recently in Sadler and Jerolmack^27^. Rate data in the compilation are all measured from terrestrial alluvial deposits, and no distinction is made on the basis of lithology/sediment type or environmental setting. All rates are quantified as a sediment thickness divided by the timespan over which the sediment was deposited. Accumulation rates are measured at timespans ranging across 10 orders of magnitude, from minutes to 44 k.y. Location data (decimal latitude and longitude) are taken from the original publications, or deduced by cross-referencing of maps/location names with Google Earth where necessary. Where multiple rates exist within a small area (<~1 km^2^) and from a similar environmental setting, an averaged location is used. A full listing of all data and publications is provided in Supplementary Data 1.

#### Rate measurement methods

We distinguish two main data types: directly measured rates and geologic rates. Directly measured rates are those that are based on observation of active sedimentation (e.g., sediment trap data and channel depth surveys). For these data, timespans are defined by the measurement period. Geologic rates are based on calculation of the rate of deposition of a previously deposited sediment pile (e.g., floodplains, terraces). For these measurements, timespans are calculated from dates of the lower and upper bounding horizons of the sedimentary succession. For many data, the upper bounding surface is the land surface. For these data, the upper date is taken as the date of publication unless information on when fieldwork occurred is provided by the authors. Absolute geologic dating methods used to constrain timespans are radiocarbon (‘^14^C’) and optically stimulated luminescence (‘OSL’) (see Fig. 3 for a listing of all measurement methods). Other dates are provided by stratigraphic markers of known age (‘Stratigraphy’). Such markers include ash beds from known volcanic eruptions, or other lithological markers that can be unambiguously related to a historic event such as soil type changes related to dam construction or land use changes (e.g., ref. ^37^). Dates based on ^137^Cs abundance (‘^137^Cs’) are considered separately, and rely on attribution of a peak or peaks in ^137^Cs abundance to nuclear bomb test events in the 1940s to 1960s (e.g., ref. ^38^). Archeological dates (‘Archeaology’) are based on dating of artefacts, such as coin finds (e.g., ref. ^39^). Dendrochronological dates (‘Dendrochronology’, e.g., ref. ^40^) are based on measuring sediment thicknesses overlying buried tree roots, with the timespan of measurement calculated from dendrochronologically defined tree age. In the case of rates constrained by two differing methodologies, the lower (older) dating method is used to classify the measurement method.

#### Age calibration

The ages of accumulation rate data in the compilation are taken from the original published sources, and for directly measured rate data (i.e., recent rates of active sedimentation) ages will be half the measured timespan, plus the time interval from the final date of measurement to the present day (2019). Where precise dates of measurement are not provided, this date is set to the publication date. Thus, a source of error in these data will thus be the possible time lag between data collection and publication. For geologic rates of sedimentary piles that do not extend to the surface, ages will be half the timespan plus the time interval between the upper bounding date and the present day.

Radiocarbon (‘^14^C’) dates dominate our data (53% of rates), and are from publications spanning from the 1960s to the present day—an interval that encompasses significant change in the accuracy and precision of ^14^C dating. Anomalous ^14^C dates in the publications were rejected. These were typically anomalously old and out of stratigraphic sequence, and likely caused by “old wood” bias^29^ (see also Bias analysis, below). All ^14^C dates used in our compilation were calibrated using the northern hemisphere IntCal13 calibration curve^41^ using the CALIB program (http://calib.org) available at http://calib.org (accessed 10/07/20). By convention, calibrated ages are reported relative to 1950 CE, and thus ages were adjusted to 2019 by adding 69 to make the ages comparable with the other data. Radiocarbon ages reported as “modern” were set to 69 y to yield the most conservative rate estimates.

#### Climate and elevation data

Elevation data for each study site were mined using the GPS Visualizer website at http://www.gpsvisualizer.com (accessed 10/07/20). Mean annual precipitation and mean annual temperature data for each study site are for the period 1970–2000 and are from the WorldClim 2.0 database^42^.

### Statistical methods

#### Monte Carlo error modeling

We accounted for uncertainties in our data using a Monte Carlo modeling approach. To do this, we generated 10,000 simulations of the compilation that had errors added to the timespan data. Rates and ages were then re-calculated, and Wilcoxon rank sum testing was carried out on each of the simulations following the same approach used for the raw data (outlined in “Rank sum testing”, below). Timespan errors were drawn from sets of normally distributed random numbers, with the size of the error scaled depending on the timespan dating method. For most methods of dating, errors scale with age. Errors in ^14^C dates in the compilation were typically <10%, although this increases slightly after age calibration. To account for the fact that many rates in the compilation are constrained by both an upper and lower ^14^C age, we set the error level at 25% (1 r.s.d., relative standard deviation). This error level helps to account for even the most imprecise dates in the compilation, many of which predate accelerator mass spectrometry. Errors in OSL dates were comparable (~10%, e.g., refs. ^43,44^), and the same 25% error level was applied to these data. Rates determined using ^137^Cs, archeological finds and stratigraphic markers rely on accurate assignment of markers to specific historical dates. Most of the data associated with these methods have an upper date at the sediment surface, and errors were set at 15% (e.g., ref. ^44^). Dendrochronology dates have the potential to be highly accurate^40^ but a conservative approach was adopted and these were also simulated with 15% error to reflect possible tree ring counting errors. Directly measured rates rely on accurate surveying and record keeping, and should have negligible error. The timespan errors in these data were set at 5%.

#### Bias analysis

An important possible source of bias in our ^14^C-determined rate data is the “old wood” problem^29^. This bias can cause an overestimation of ages for alluvial strata because the dated wood within the strata reflects the age of growth rather than the (later) age of deposition. A similar issue linked to this is the possible reworking of older, previously deposited wood material via alluvial processes. Because ^14^C rates dominate pre-settlement data in the compilation (Supplementary Fig. 1), these biases could ostensibly limit pre-settlement rates, and theoretically account for the difference in rates we observe between pre- and post-settlement ages.

We tested for this bias by removing from our compilation all rate data constrained by a basal ^14^C date and a contemporary upper date at the land surface (see Rate measurement methods above). Rate data constrained by both a lower and an upper ^14^C date would not, theoretically, be prone to bias since any systematic over-estimation of age for both horizons would not affect the calculated time span (although ages may be overestimated). Analysis of these “surface-free” ^14^C rates does not reveal any significant underestimation of ^14^C rates linked to the “old wood” problem. Indeed, we actually find that pre-settlement rates are slightly slower if ^14^C rates that have an upper date at the land surface are removed from the compilation (Supplementary Fig. 2). This is because although most of these dates have a pre-settlement age, a significant fraction of the timespan of these data encompasses the post-settlement interval (because they reach the land surface). This then biases the rate toward slightly higher values. Overall, however, removing these rates reduces the size of the compilation and its statistical robustness, and we thus retain all ^14^C rates to derive our primary results.

The data in the compilation occupy a wide range of elevations, hydroclimate regimes, basins, and catchments (elevations: 0–3001 m, modern mean annual precipitation rates: 80–2482 mm y^−1^, modern mean annual temperatures: −3–23°C; see Supplementary Fig. 3). We investigated elevation and climate biases in our compilation with rank sum testing to compare mean annual precipitation, mean temperature, and elevation from pre- and post-settlement study sites. No consistent statistically significant differences were found (Supplementary Table 2). However, we note that sites containing post-settlement rates tend to have higher modern precipitation than sites containing pre-settlement rates, and at some timespans the differences are significant (*p* values <0.05, Supplementary Table 2). We investigated whether this affects our results by removing all post-settlement rates measured from sites with precipitation >1000 mm y^−1^. This removes the statistically significant differences in precipitation between pre- and post-settlement data. Rank sum analysis of the rates in this edited data set shows that our key results are unaffected, and post-settlement alluvium accumulation rates are still faster than pre-settlement rates at all comparable timespans (mean difference >10×, *p* values <0.0005). Overall, our results are unlikely to be biased by differences in elevation, climate or precipitation between pre- and post-settlement rate data.

#### Rank sum testing

Wilcoxon rank sum tests were used to test for difference of medians between pre- and post-settlement rate data at comparable timespans. Testing was carried out in Matlab using the ranksum function on data divided into 0.2 log timespan bins. This test (equivalent to a Mann–Whitney *U* test) does not assume normally distributed data or demand data sets of equal size. *P* values output by these tests are the probability that the data being compared come from continuous distributions with equal medians. Specifically, a one-sided test is used to test whether post-settlement medians are faster than pre-settlement medians. In Supplementary Table 2 (which shows the results of investigations into climate and elevation bias), two-sided rank sum tests were used because the aim was to establish only if post-settlement medians were different (lower or higher) than pre-settlement medians.

#### Change detection analysis

Two methods were used to corroborate the visual indication from Fig. 2 that rates of alluvium accumulation increased significantly at an age of ~200 y. Change-point analysis determines the position within a time series where the mean value changes most significantly. The data are partitioned in such a way that the sum of the residual (squared) error of each segment from its local mean is minimized. This analysis was implemented using the findchangepts function in Matlab on LOESS smoothed versions of the rate versus age data in order to avoid change-points that arise from clusters of anomalously low or high values rather than systematic (i.e., stepwise) change. For smoothing intensities between 30% and 90%, the two most significant change-points in the data occur between ~100 and ~300 y. A sliding window Wilcoxon rank sum test was also used that followed the methods outlined above, but with the analysis carried out within paired (contiguous) windows of fixed length that are incrementally moved along the unsmoothed age versus rate data. The age in the data set where minimum *p* values are reached (i.e., where rates within each paired window are least likely to come from a continuous distribution with equal median) is between 190 and 245 y for window sizes of 200–1000 data points.

## Supplementary information

Supplementary information

Description of Additional Supplementary Files

Supplementary Data 1

## Data Availability

All alluvium accumulation rate data, and a publication reference list for these data, are provided in Supplementary Data 1.
