# Supplementary material for: The human impact on North American erosion, sediment transfer, and storage in a geologic context
Source: Nat Commun. 2020 Nov 26;11:6012. doi: 10.1038/s41467-020-19744-3 (PMC7691505; doi:10.1038/s41467-020-19744-3)
Supplement: Supplementary file 1 — Supplementary information [file 41467_2020_19744_MOESM1_ESM.pdf]

SUPPLEMENTARY INFORMATION FOR:

The human impact on North American erosion, sediment transfer and storage in a geologic context

David B. Kemp, Peter M. Sadler and Veerle Vanacker

Log timespan bin	Approx. timespan range (y)	Pre-settlement rates (>300 y old)			Post-settlement rates (<300 y old)			Raw rank sum p-value	% of significant Monte Carlo sims. (p-value <0.05)
		Number of rates	Number of study sites	Raw (and Monte Carlo) median rate (mm y^{-1})	Number of rates	Number of study sites	Raw (and Monte Carlo) median rate (mm y^{-1})		
1.6	40-63	7	4	1.21 (1.84)	123	26	15.12 (15.88)	0.021	88.8%
1.8	63-100	29	12	1.21 (1.27)	169	28	23.22 (22.61)	<0.0001	100%
2	100-158	29	11	0.68 (0.92)	196	45	5.93 (7.87)	<0.0001	100%
2.2	158-250	73	21	0.64 (0.76)	141	33	6.71 (6.88)	<0.0001	100%
2.4	250-400	151	41	0.59 (0.67)	11	8	6.15 (5.50)	<0.0001	100%

Supplementary Table 1. Wilcoxon rank sum test results that compare pre- and post-settlement rates of alluvium accumulation at timespans between ~40 and 400 y. The cutoff age used to distinguish pre- and post-settlement rates is 300 y. This contrasts with Table 1 of the main paper, where a cutoff age of 200 y was used. Wilcoxon rank sum p-values represent the probability that both pre- and post-settlement rates come from a continuous distribution with equal medians. Rank sum results are shown for both the raw data in the compilation, and from 10,000 error-prone Monte Carlo simulations of the raw data (see Methods). The percentage of simulations that yield significant differences between pre- and post-settlement rates (p-values <0.05) is also shown.

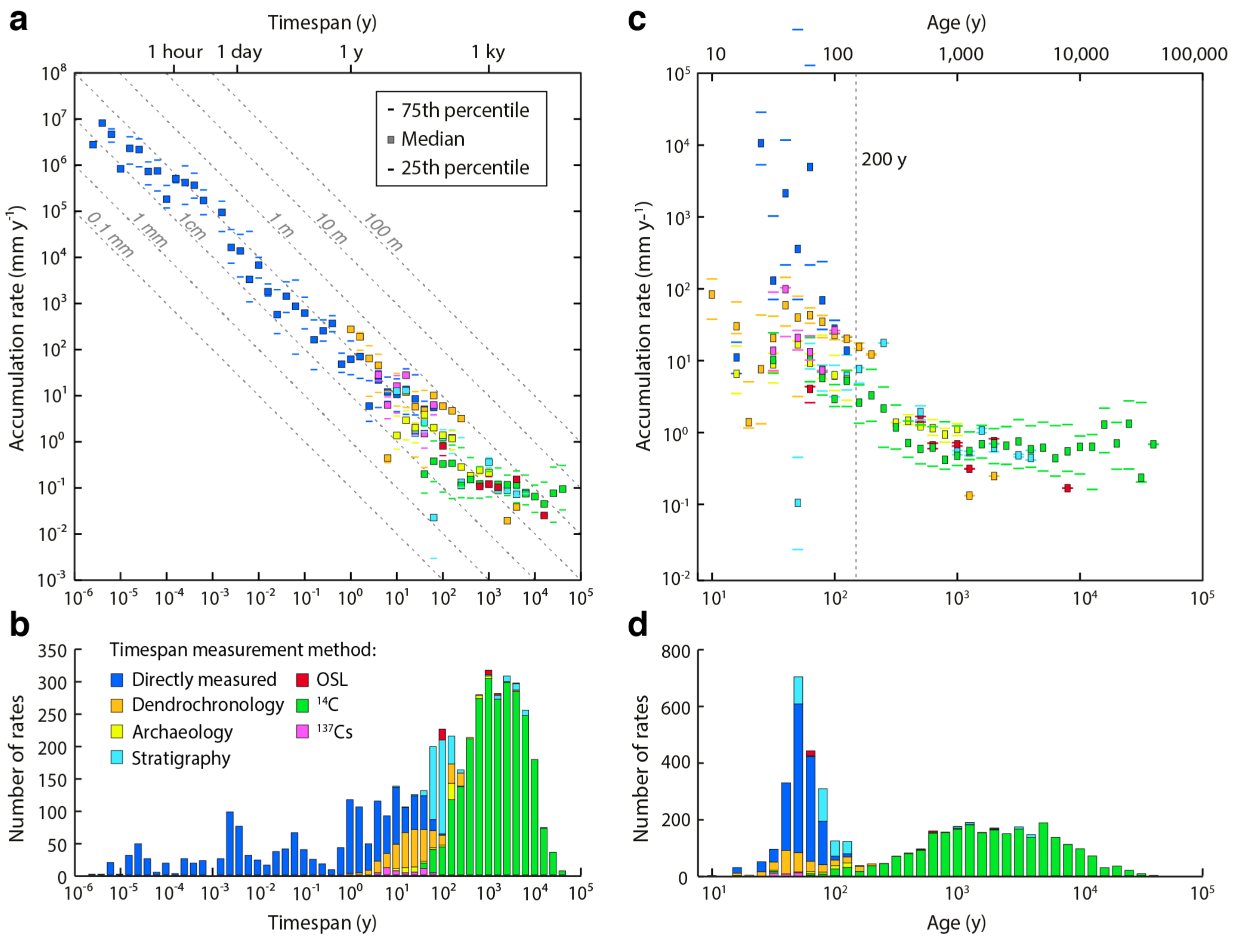

Supplementary Fig. 1. Alluvium accumulation rates plotted against timespan and age, and distinguished by measurement method. **a** Plot of median rates versus timespan, color-coded based on the timespan measurement method used to calculate rates. Median rates and the associated 25th-75th percentile range are calculated from the raw data in 0.2 log timespan bins. Dashed diagonal lines are contours of constant sediment thickness. Note how timespan dependent scaling of rates occurs regardless of measurement method, and that there are no clear differences in rates measured by different methods. **b** Histogram showing data abundance in each 0.2 log timespan bin. **c** Plot of median rates versus age. Data are binned in 0.1 log age bins. Note how the increase in accumulation rates at ~200 y is seen in rates measured using different methods. **d** Histogram showing data abundance in each 0.1 log age bin. See Methods for information on the different rate measurement methods.

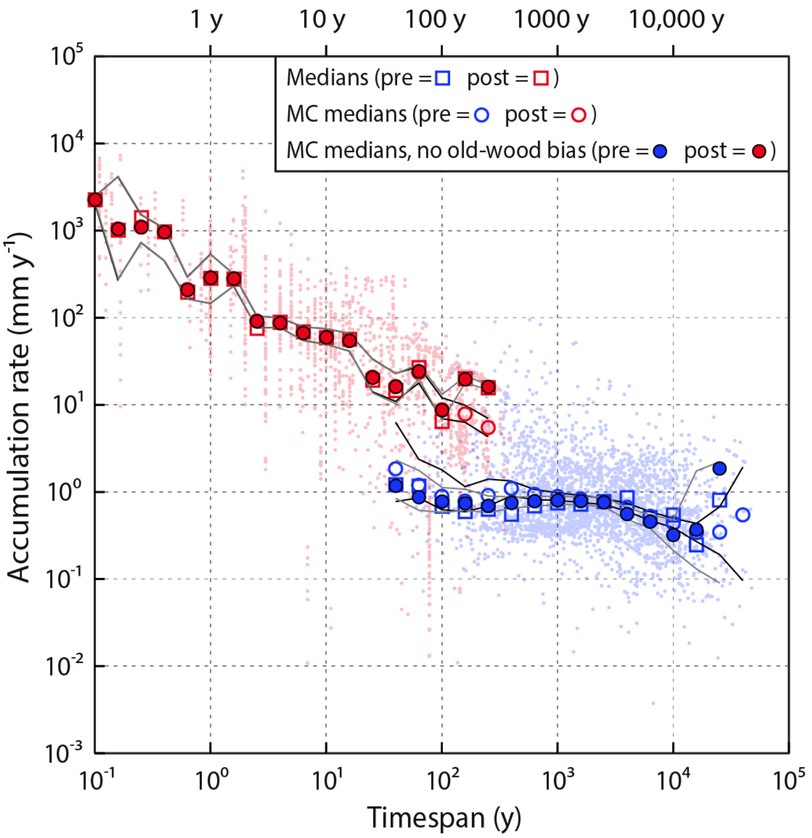

Supplementary Fig. 2. Plot of median rates of alluvium accumulation versus timespan (in 0.2 log timespan bins). Data are divided into pre-settlement (blue data, >200 y old) and post-settlement (red data, <200 y old). Small dots are the raw rate data in the compilation. Empty squares are the median rates of the raw data (measured in 0.2 log time span bins). Empty circles are the median rates calculated from Monte Carlo (MC) modelling of the raw data that takes into account rate measurement errors (see Methods). Black lines show the 95% confidence intervals associated with this error modelling. Filled circles are the median rates calculated from the same Monte Carlo modelling procedure, but with rates that are susceptible to 'old wood' bias removed prior to analysis (grey lines show the 95% confidence intervals). The rates removed are those that are constrained by a basal ^{14}C date and a contemporary upper date at the land surface. These rates may be underestimated because ^{14}C dates relate to the age of wood growth, and not necessarily the age of deposition – which may be later (see ref. 29 of main text). The removal of these potentially biased rates does not significantly alter the observation that post-settlement rates are ~10 x faster than pre-settlement rates at timespans where pre- and post-settlement rates co-occur (~40 to ~400 y) (see Methods for full details).

	Log timespan bin	Approx. timespan range (y)	Pre-settlement data		Post-settlement data		Rank sum p-value
			Number of study sites	Median	Number of study sites	Median	
MEAN ANNUAL TEMPERATURE (°C)	1.6	40-63	4	15.18	26	12.88	0.08
	1.8	63-100	12	12.39	27	12.91	0.51
	2	100-158	11	12.40	45	11.48	0.61
	2.2	158-250	21	12.40	34	10.26	0.75
	2.4	250-400	33	10.44	18	11.21	0.37
MEAN ANNUAL PRECIPITATION (MM)	1.6	40-63	4	476	26	1161.5	0.04*
	1.8	63-100	12	592	27	1186	0.09
	2	100-158	11	541	45	1025	<0.001*
	2.2	158-250	21	571	34	826	0.39
	2.4	250-400	33	571	18	1086.5	0.03*
STUDY SITE ELEVATION (M)	1.6	40-63	4	526.85	26	329.4	0.93
	1.8	63-100	12	692.1	27	220.4	0.04*
	2	100-158	11	472.7	45	212.4	0.11
	2.2	158-250	21	408	34	279.15	0.68
	2.4	250-400	33	499.4	18	186.55	0.09

Supplementary Table 2. Wilcoxon rank sum test results that compare climate and elevation between pre- and post-settlement sites in the compilation. This testing is designed to test whether environmental differences could partially explain the faster rates of post-settlement alluvium accumulation compared to pre-settlement data. Few statistically significant differences exist (p-values <0.05, marked with *). However, median precipitation is higher at sites containing post-settlement data compared to sites containing pre-settlement data. Nevertheless, as noted in the main text, removal of post-settlement rates measured at sites with high precipitation (>1000 mm y^{-1}) does not change our results (see Methods for full details and data sources).

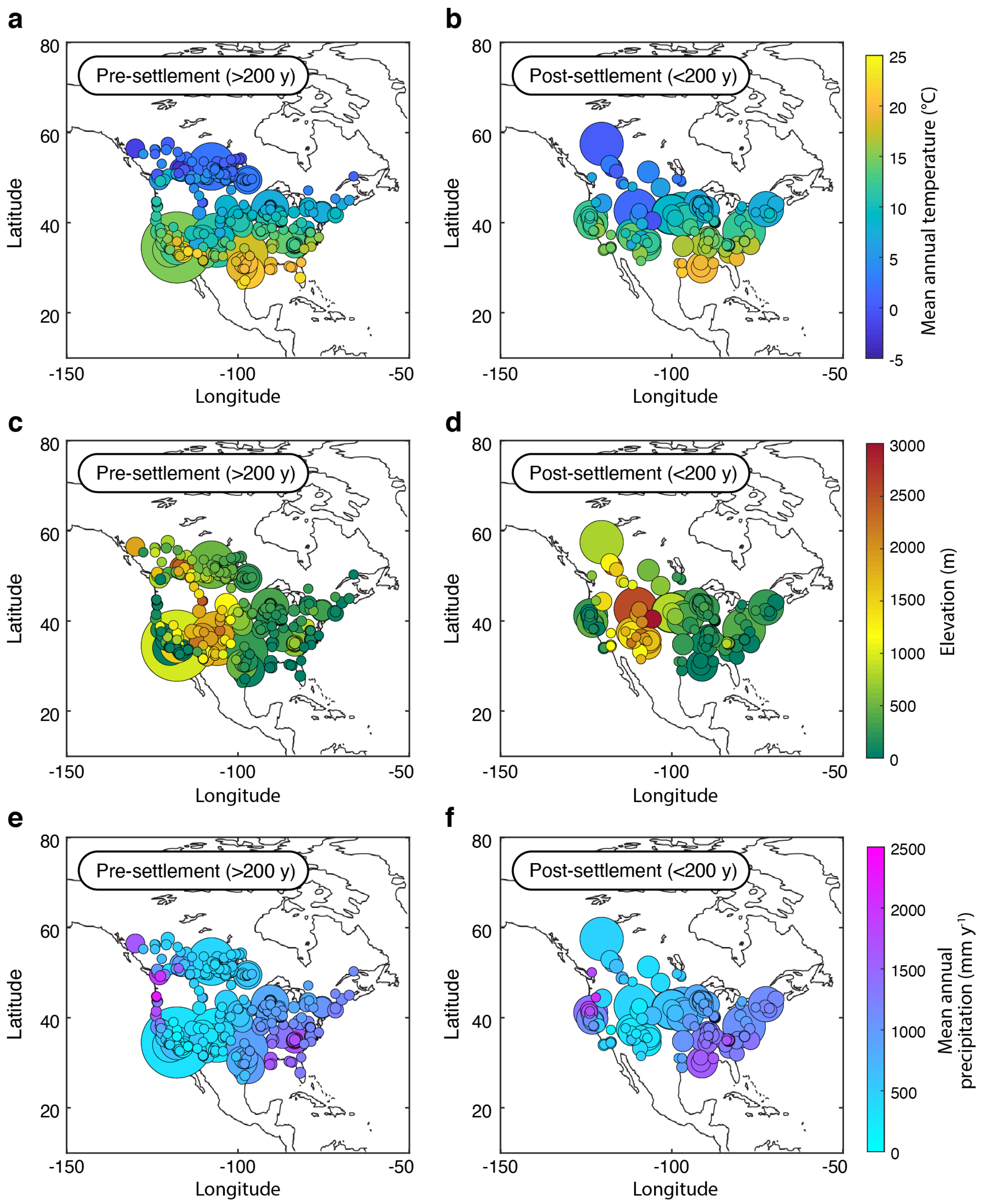

Supplementary Fig. 3. Maps showing distribution of pre- and post-settlement accumulation rates in the compilation. Data are colour coded by mean annual temperature (**a**, **b**), site elevation (**c**, **d**) and mean annual precipitation rate (**e**, **f**). Note the generally even distribution of data, and similar ranges in elevation, precipitation and temperature for pre- and post-settlement study sites. See Methods for data sources.
