# Supplementary material for: The human impact on North American erosion, sediment transfer, and storage in a geologic context
Source: Nat Commun. 2020 Nov 26;11:6012. doi: 10.1038/s41467-020-19744-3 (PMC7691505; doi:10.1038/s41467-020-19744-3)
Supplement: Supplementary file 2 — Description of Additional Supplementary Files [file 41467_2020_19744_MOESM2_ESM.pdf]

Description of Additional Supplementary Files

File Name: Supplementary Data 1
Description: Excel spreadsheet containing: 1) table of all alluvium accumulation rate data, and 2) publication reference list for these data.
